# THE EFFECT OF COMMUNITY HEALTHCHOICES ON USE OF HOME AND COMMUNITY-BASED SERVICES

**DOI:** 10.1093/geroni/igac059.563

**Published:** 2022-12-20

**Authors:** Keri Kastner

**Affiliations:** University of Pittsburgh, Pittsburgh, Pennsylvania, United States

## Abstract

Medicaid claims data for the years 2013 to 2020 were analyzed to examine use of common categories of home and community-based services (HCBS) as well as the proportion of people living in nursing homes as opposed to receiving long-term services and supports in the community. There was a long-term trend prior to Community HealthChoices of a shift the locus of LTSS away from nursing homes. The implementation of managed care to deliver LTSS continued, but did not appear to accelerate this trend. However, MLTSS did appear to control to growth in hours of personal care per person both in the aggregate and longitudinally within the same individuals over time. There were decreases in the use of adult day services and home delivered meals. However, the decline in home delivered meals was more than offset by an increase in uptake of the supplementary nutritional assistance program (SNAP).

